# Molecular Mechanism of Rectification at Identified Electrical Synapses in the *Drosophila* Giant Fiber System

**DOI:** 10.1016/j.cub.2008.10.067

**Published:** 2008-12-23

**Authors:** Pauline Phelan, L. Ann Goulding, Jennifer L.Y. Tam, Marcus J. Allen, Rebecca J. Dawber, Jane A. Davies, Jonathan P. Bacon

**Affiliations:** 1 Department of Biosciences, University of Kent, Canterbury CT2 7NJ, UK; 2 Department of Biology and Environmental Science, School of Life Sciences, University of Sussex, Brighton BN1 9QG, UK

**Keywords:** MOLNEURO

## Abstract

Electrical synapses are neuronal gap junctions that mediate fast transmission in many neural circuits [1–5]. The structural proteins of gap junctions are the products of two multigene families. Connexins are unique to chordates [3–5]; innexins/pannexins encode gap-junction proteins in prechordates and chordates [6–10]. A concentric array of six protein subunits constitutes a hemichannel; electrical synapses result from the docking of hemichannels in pre- and postsynaptic neurons. Some electrical synapses are bidirectional; others are rectifying junctions that preferentially transmit depolarizing current anterogradely [11, 12]. The phenomenon of rectification was first described five decades ago [1], but the molecular mechanism has not been elucidated. Here, we demonstrate that putative rectifying electrical synapses in the *Drosophila* Giant Fiber System [13] are assembled from two products of the innexin gene *shaking-B*. Shaking-B(Neural+16) [14] is required presynaptically in the Giant Fiber to couple this cell to its postsynaptic targets that express Shaking-B(Lethal) [15]. When expressed in vitro in neighboring cells, Shaking-B(Neural+16) and Shaking-B(Lethal) form heterotypic channels that are asymmetrically gated by voltage and exhibit classical rectification. These data provide the most definitive evidence to date that rectification is achieved by differential regulation of the pre- and postsynaptic elements of structurally asymmetric junctions.

## Results and Discussion

### Phenotypic Rescue of *shakB^2^* Mutants: *shakB(n+16)*, and Not *shakB(n)*, Is Required in the Giant Fiber

**Figure 1:**
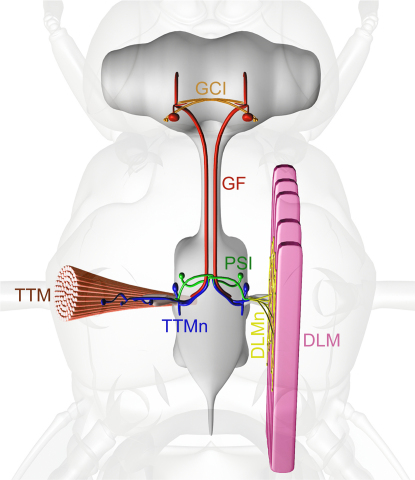
Neurons and Muscles of the *Drosophila* Giant Fiber System The Giant Fiber System mediates escape behavior in the fly. Visual stimuli activate a pair of large interneurons, the Giant Fibers (GF, red). In the brain, the GFs form synaptic connections with the giant commissural interneurons (GCI, orange). The GF axons descend to the mesothoracic ganglion, where they terminate in characteristic lateral bends. The GF bends form extensive synaptic connections with the medially directed dendrites of the motorneurons (TTMn, blue) of the tergotrochanteral (jump) muscles (TTM, brown; left) of the middle leg. Just before the bends, the GF axons synapse with the peripherally synapsing interneurons (PSI, green), which, in turn, innervate the motorneurons (DLMn, yellow) of the dorsal longitudinal (flight) muscles (DLM, pink; right). The PSI axons and terminal tips of the TTMn medial dendrites synapse with one another at the midline. The central synapses are electrical or mixed electrochemical synapses. The neuromuscular junctions, which, for clarity, are shown on one side only of the bilaterally symmetrically pathway, are chemical synapses. Reprinted from [13] with permission from Elsevier.

The *shaking-B* (*shakB*) gene gives rise to several partially identical transcripts, which translate into three distinct proteins: Shaking-B(Neural) (ShakB[N]) [16], Shaking-B(Neural+16) (ShakB[N+16]) [14], and Shaking-B(Lethal) (ShakB[L]) [17]. ShakB(N) was originally implicated in synaptic connectivity in the Giant Fiber System (GFS) (Figure 1). The mutation *shakB^2^*, believed to lie in an exon unique to the *shakB(n)* transcript [16], was associated with loss of electrical and dye coupling [18–20] and gap-junction morphology [21] at GFS synapses. ShakB(N+16) subsequently was found to be partially encoded on this exon [14]; thus, *shakB^2^* disrupts the function of ShakB(N) and ShakB(N+16). To determine directly which of these proteins is required at electrical synapses in the GFS, we sought to rescue the mutant phenotype by cell-specific expression of the individual transcripts under GAL4-UAS control [22]. The GAL4 lines *A307*, which expresses strongly in the Giant Fibers (GFs) and giant commissural interneurons (GCIs) and weakly in the tergotrochanteral muscle motorneurons (TTMns) and peripherally synapsing interneurons (PSIs) (Figure 1) [20, 23], and *c17*, which expresses in the GFs, but not in its pre- or postsynaptic partners (Figure 1) [24], were used to direct expression of UAS*-shakB(n+16)* or UAS*-shakB(n)* to neurons of the GFS in a *shakB^2^* mutant background.

**Figure 2:**
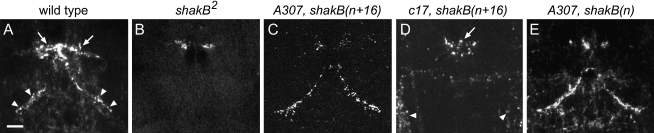
ShakB Immunolabeling Is Restored at GFS Synapses in Transgenic *shakB^2^* Flies Corresponding regions of the mesothoracic neuromere of the adult CNS labeled with antisera that detect all ShakB proteins. Images are projections of confocal z series. (A) Wild-type. The midline anterior cluster of staining (arrows) corresponds to the region of contact between axons of the GFs and PSIs and the tips of the TTMn medial dendrites. More posteriorly, the bilateral tracts of staining (arrowheads) profile regions of contact between the terminal bend of the GF axon and the medial dendrite of the TTMn. (B) *shakB^2^* mutants. Immunolabeling is abolished with the exception of discrete spots at the region of the GF-PSI-TTMn contacts. (C–E) Transgenic mutant lines. *A307*-GAL4-directed (C and E) or *c17*-GAL4-directed (D) expression of UAS-*shakB(n+16)* (C and D) and UAS-*shakB(n)* (E) transgenes in a *shakB^2^* mutant background restores ShakB expression at GFS synapses. In *A307* transgenic lines (C and E), immunolabeling is restored at the GF-TTMn and, to a lesser extent, at the GF-PSI synapses. In *c17* transgenic lines (D), labeling is restored at the GF-PSI-TTMn midline contacts, but not, to detectable levels, at the GF-TTMn synapses. Arrowheads in (D) indicate non-GFS sensory neurons known to express *c17*[29]. The genotypes of transgenic flies are as follows: (C) *shakB^2^; A307*-GAL4/UAS-*shakB*(*n+16)*, (D) *shakB^2^; c17*-GAL4/UAS-*shakB*(*n+16*) or *shakB^2^; c17*-GAL4/*c17*-GAL4; UAS-*shakB*(*n+16*), and (E) *shakB^2^; A307-*GAL4; UAS*-shakB(n)*. The scale bar represents 10 μm and applies to all panels.

Figure 2 compares ShakB protein distribution in nervous systems of wild-type, *shakB^2^*, and transgenic mutant flies. The wild-type pattern of immunolabeling (Figure 2A) was undetectable at GF-TTMn synapses in *shakB^2^* mutants; some labeling persisted in the region of GF-PSI-TTMn contacts (Figure 2B), presumably reflecting expression of ShakB(L), which is unperturbed in *shakB^2^* (see below). *A307*-directed expression of *shakB(n+16)* (Figure 2C) or *shakB(n)* (Figure 2E) in a *shakB^2^* background restored immunofluorescence at the GFS synapses. The intensity of staining at GF-TTMn synapses was comparable to that in wild-type, whereas levels at the GF-PSI-TTMn contacts were consistently lower than in wild-type. ShakB protein distribution in the brain [20] was also restored in *A307* transgenic flies (data not shown). *c17*, which is a much weaker GF driver than *A307* [24], did not restore ShakB(N+16) expression in *shakB^2^* mutants to detectable levels at GF-TTMn synapses (Figure 2D), although phenotypic rescue studies (see below) implied the presence of a low level of protein. It was possible to detect expressed protein at the midline synapses (Figure 2D, arrow) possibly because it was less diffusely distributed at these discrete junctions than at the more extensive GF-TTMn contacts (see Figure 1).

**Figure 3:**
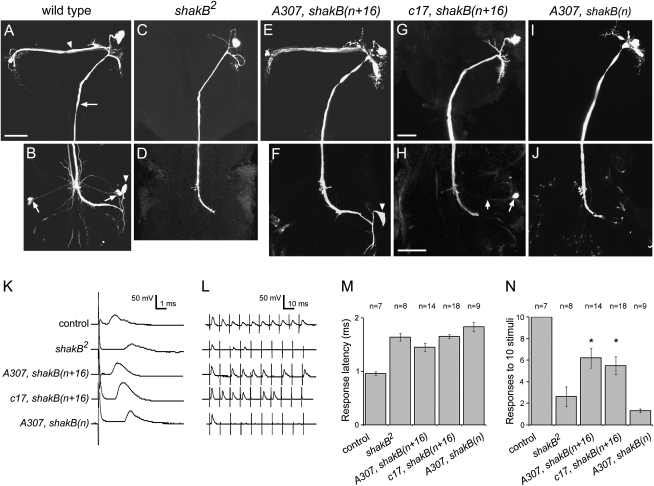
Targeted Expression of *shakB(n+16)*, but Not *shakB(n)*, Rescues Dye Coupling and Synaptic Activity in the GFS of *shakB^2^* Mutants (A–J) Dye coupling between neurons of the GFS. Lucifer yellow was injected into one GF axon and visualized directly (A–E, G, and I) or after labeling with antilucifer yellow antibody (F, H, and J). Images are projections of confocal Z series. (A and B) Wild-type. Lucifer yellow diffuses from the injected GF (A, arrow) into the GCIs in the brain (A, arrowhead) and into the ipsilateral TTMn (B, arrowhead), both PSIs (B, arrows), and other neurons in the mesothoracic ganglion. Weak coupling to the contralateral GF is also observed. (C and D) Dye coupling in the brain (C) and thoracic ganglion (D) is eliminated in *shakB^2^* mutants. (E and F) *A307*-directed expression of *shakB(n+16)* in *shakB^2^* mutants restores coupling to the GCIs (E) and the TTMn (F, arrowhead). (G and H) *c17*-directed expression of *shakB(n+16*) in *shakB^2^* mutants rescues coupling to the PSI (H, arrows). (I and J) Expression of *shakB(n)* in *shakB^2^* mutants with *A307* fails to restore dye coupling to neurons in the brain (I) or mesothoracic ganglion (J). Scale bars represent 40 μm; (B–F), (I), and (J) are as for (A). (K–N) Recordings from the TTM muscle in response to GF simulation. (K and L) Traces from individual flies showing (K) response latency to a single stimulus and (L) responses to a train of ten stimuli delivered at a frequency of 100 Hz. In controls, response latency is short (<1 ms), and ten out of ten stimuli elicit a response. *shakB^2^* mutants exhibit long latency responses and poor following frequency. *A307*- or *c17*-directed expression of *shakB(n+16)* in *shakB^2^* mutants partially rescues response latency in some flies and increases the response to repetitive stimuli in most flies. Rescue is never observed in *shakB^2^* mutants expressing *shakB(n)* under the control of *A307*. (M and N) Mean ± SEM data for response latency (M) and following frequency (N) for n flies recorded. ^∗^p < 0.05 in unpaired Student's t test. Genotypes of transgenic flies are as in Figure 2. In (K)–(N), controls are *shakB^2^*/+; UAS-*shakB(n+16)*; *shakB^2^* are *shakB^2^*/Y; UAS-*shakB(n+16)*.

Gap-junction function in the GFS was examined by monitoring the cell-cell transfer of the fluorescent dye Lucifer Yellow injected into one of the GF axons [20] (Figures 3A–3J and Table S1 available online). In wild-type flies, the dye diffused from the injected GF into the GCIs in the brain (Figure 3A), the TTMn, PSI, and other unidentified neurons in the thoracic ganglion (Figure 3B). Dye coupling was never observed in *shakB^2^* (Figures 3C and 3D). *A307*-directed expression of *shakB(n+16)* in these mutants rescued coupling between the GF and GCIs (Figure 3E) and between the GF and TTMn (Figure 3F); rescue of GF-PSI coupling was observed less frequently (Table S1), consistent with the comparatively lower levels of expressed protein at these junctions (Figure 2C). Expression of *shakB(n+16)* under the control of *c17* did not rescue GF-GCI coupling in *shakB^2^* (Figure 3G). In the thoracic ganglion, coupling between the GF and PSI was rescued; however, GF-TTMn dye coupling was not convincingly rescued (Figure 3H). These data are consistent with the finding that ShakB protein was more concentrated at the GF-PSI contacts than at the GF-TTMn contacts in these flies (Figure 2D). In ∼24% of preparations, we did observe very faint fluorescence in a cell in the approximate position of the TTMn. We reasoned, therefore, that there was weak rescue at both synapses but that the dye dissipated in TTMn, which is a much larger cell than PSI. To confirm whether this was the case, we used an alternative method to assess GFS synaptic function.

Electrophysiological recordings were made from the tergotrochanteral (TTM) and dorsal longitudinal (DLM) muscles in response to GF stimulation [24] (Figures 3K–3N and Table S2). Using this approach, rescue of the GF-TTM pathway was observed when *shakB(n+16)* was expressed in *shakB^2^* mutants with either *c17* or *A307*. The level of rescue obtained was slightly higher with *A307* but, in both cases, was manifest as a dramatic increase in the number of flies responding (Table S2), a slight (although not statistically significant) reduction in response latency (Figures 3K and 3M), and a significant improvement in the response to repetitive stimulation at 100 Hz, indicative of more stable synapses (Figures 3L and 3N and Table S2).

Expression of *shakB(n)* in *shakB^2^* mutants with the stronger GAL4 driver *A307* failed to restore dye coupling (Figures 3I and 3J and Table S1) or synaptic activity (Figures 3K–3N and Table S2) in the GFS, although the protein was clearly localized to sites of synaptic contact (Figure 2E).

### GFS Synapses Are Assembled from Two Products of the *shakB* Gene

The phenotypic rescue studies demonstrate that *shakB(n+16)* is required for electrical transmission in the GFS and that *shakB(n)*, despite sharing 96% amino acid identity [14], cannot functionally substitute.

The use of two GAL4 lines, with different patterns of expression in the GFS, to drive *shakB(n+16)* allowed us to determine which cells of the escape circuit require this protein. Robust rescue of the GF-GCI synapses was observed with *A307*, which expresses strongly in both of these neurons, but not with *c17*, which expresses in the GFs only. This suggests that the protein is normally required in both cells. Synapses between the GF and its thoracic ganglion targets TTMn and PSI were rescued when *shakB(n+16)* was expressed with either *A307* or *c17*. In principle, rescue with A307 could be due to formation of ShakB(N+16) homotypic channels because this GAL driver expresses in the TTMn and PSI as well as in the GF. In practice, postsynaptic expression of the transgene is unlikely to have contributed significantly to synaptic connectivity because the rescue lines are heterozygous for the GAL4 construct, and *A307* expression (e.g., of reporter genes) in TTMn and PSI is weak even in the homozygous state. Rescue with *c17*, which does not express in TTMn or PSI, confirms that coupling between the GF and these cells is not dependent on the presence of *shakB(n+16)* postsynaptically.

We conclude that the synapses between the GF and GCIs, which are believed to synchronize activity of right and left GFs [20], are homotypic gap junctions with both pre- and postsynaptic hemichannels composed of ShakB(N+16). The GF-TTMn and GF-PSI synapses are heterotypic junctions in which presynaptic ShakB(N+16) interacts with a different innexin in the postsynaptic neurons. Consistent with these data, *shakB(n+16)* has been shown by RNA in situ hybridization to be expressed in the wild-type GFs and in the presumptive GCIs; it is the only *shakB* transcript detectable in these neurons and is not present at detectable levels in the TTMn or PSI (Figure S1) [14, 17]. Two lines of evidence indicate that these cells express *shakB(l)*. First, a GAL4 construct containing the *shakB(l)* promoter, which is distinct from the *shakB(n+16)* promoter, drives reporter gene expression in TTMn and PSI (but not in the GFs) (Figure S1) [15]. Second, ShakB immunoreactivity persists in *shakB^2^* mutants at the midline in the region where the PSI contacts the tip of the TTMn medial dendrite and the GF (Figure 2B); given the specificity of the antibody, this must represent ShakB(L) protein. A caveat is that *shak-B(l)* RNA is not detectable in the TTMn or PSI by in situ hybridization, presumably because expression levels are below the sensitivity of the technique.

Since the pioneering work of Furshpan and Potter [1], several studies have examined the mechanism of rectification at the crayfish giant motor synapse, the classical rectifying electrical synapse [25, 26], where transmission can be recorded by inserting electrodes into the pre- and postsynaptic axons. Ideally, one would like to apply the same approach in the *Drosophila* GFS in order directly to correlate synaptic physiology and molecular genetics. As yet, at least, this is not technically possible because the fruit fly neurons are much smaller in size and less accessible than their crayfish counterparts. To determine, therefore, whether molecular asymmetry at the GFS synapses might underlie electrical rectification, we “modeled” a synapse in paired *Xenopus* oocytes.

### Functional Expression in *Xenopus* Oocytes: ShakB(N+16) and ShakB(L) Form Heterotypic Channels with Rectifying Properties

**Figure 4:**
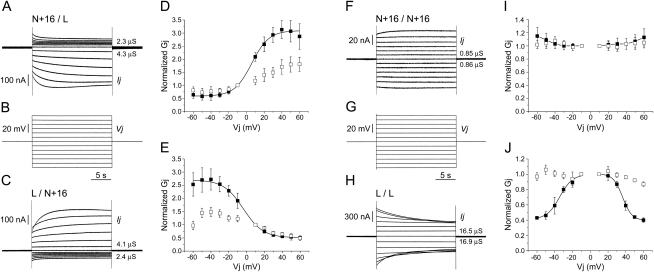
ShakB(N+16) and ShakB(L) Form Heterotypic Channels that Are Asymmetrically Gated by Voltage, Whereas Homotypic Channels Exhibit Symmetrical Voltage Responses *Xenopus* oocytes injected with *shakB* RNAs were paired and recorded by dual voltage clamp. Both cells of a pair were clamped at a holding potential of −80 mV. Transjunctional voltage steps (Vj, mV) were then applied to one cell while the current (Ij, nA) required to maintain the other cell at the holding potential was recorded. Junctional conductance (Gj, μS) is Ij/Vj. (A–E) Heterotypic cell pairs. (A–C) Representative traces from cell pairs injected with 0.25 ng RNA. Junctional currents (A and C) were elicited by application of Vj steps (B) to (A) the ShakB(N+16)-expressing cell or (C) the ShakB(L)-expressing cell. Gj is shown for depolarizing (below baseline) and hyperpolarizing (above baseline) 10 mV steps. Mean values are provided in Table S3. (D–E) Gj/Vj plots. Initial (open symbols) and steady-state (closed symbols) Gjs recorded in response to application of Vj steps to (D) the ShakB(N+16)-expressing cell or (E) the ShakB(L)-expressing cell. Gjs, normalized to their values at −10 mV (D) and 10 mV (E), are mean ± SD for n = 4 pairs injected with 0.1–0.25 ng RNA. Steady-state data are fitted to a Boltzmann equation (parameters in Table S3). Heterotypic channels respond asymmetrically to applied voltage. (F–J) Homotypic cell pairs. (F–H) Typical recordings and Gj/Vj plots (I and J) for oocyte pairs in which both cells expressed (F and I) ShakB(N+16) (0.5–2 ng RNA) or (H and J) ShakB(L) (0.05–0.25 ng RNA). (F and H) Gj is shown for depolarizing and hyperpolarizing 10 mV steps. (I and J) Initial (open symbols) and steady-state (closed symbols) Gjs normalized to their values at Vj = ±10 mV are mean ± SD for n = 8 (I) and n = 3 (J) pairs. Curves in (J) are Boltzmann fits of the data. ShakB(N+16) channels show no significant voltage sensitivity. ShakB(L) channels exhibit a symmetrical response to applied voltage. Mean Gjs and Boltzmann parameters are in Table S3.

*shakB(n+16)* and *shakB(l)* RNAs were transcribed in vitro and microinjected into connexin-depleted *Xenopus* oocytes [6, 27]. Figure S2 confirms that both RNAs are efficiently translated by oocytes. The ability of the expressed proteins to form channels was assessed by dual voltage clamp electrophysiology [28] of cell pairs in which one cell expressed ShakB(N+16) and the other ShakB(L) (heterotypic) or both cells of a pair expressed the same protein (homotypic). In heterotypic configuration, channels were reliably induced at RNA levels of 0.1–0.5 ng; the voltage sensitivity of these channels differed significantly from that of homotypic channels composed of either protein (Figure 4 and Table S3).

A striking asymmetry was observed in the response of ShakB(N+16)/ShakB(L) heterotypic channels to transjunctional voltage (Vj). Depolarizing Vj steps applied to the ShakB(N+16)-expressing cell induced large junctional currents (Ijs) that tended to increase over time for Vjs up to 40 mV. For higher Vjs, current increased to its maximum level and then declined slightly (Figures 4A and 4B). By contrast, when the ShakB(L)-expressing cell was subjected to depolarizing Vjs, induced currents were of low magnitude and showed a voltage-dependent decrease over time (Figures 4B and 4C). Hyperpolarizing Vjs applied to either cell elicited responses opposite to those observed on depolarization. Junctional currents induced by the application of negative Vjs to the ShakB(N+16)-expressing cell were of low magnitude and decreased in a time- and voltage-dependent manner (Figures 4A and 4B). Large currents were induced when hyperpolarizing Vjs were applied to the ShakB(L)-expressing cell. These Ijs increased over time to a steady-state level for Vjs up to 40 mV; for higher Vjs, maximum Ij was followed by a slight decline (Figures 4B and 4C).

Figures 4D and 4E show the relationship between junctional conductance (Gj) and transjunctional voltage for ShakB(N+16)/ShakB(L) heterotypic channels. Conductance was low and declined with increasing Vj (up to 40–50 mV) for relative negativity of the ShakB(N+16)-expressing cell (Figure 4D, left half of the graph) or for relative positivity of the ShakB(L)-expressing cell (Figure 4E, right half of the graph). Instantaneous Gj, measured 5 ms after the imposition of Vj steps, was not significantly different than steady-state Gj, indicating that the junctions approximated their lowest conductance state, which was always > 0, within 5 ms. The residual conductance presumably represents a small population (∼15%–∼20%) of voltage-insensitive channels. Instantaneous and steady-state Gj increased in a sigmoidal fashion as the cell expressing ShakB(N+16) was depolarized (Figure 4D) or the ShakB(L)-expressing cell was hyperpolarized (Figure 4E) relative to its heterotypic partner. Steady-state data fitted well to a Boltzmann equation, suggesting a single transition from closed to open states. The calculated parameters were essentially the same irrespective of which cell of the pair was subjected to voltage steps (Table S3).

The asymmetry of the voltage response of heterotypic channels contrasts with a symmetrical response of homotypic channels to applied voltage. ShakB(N+16) channels, which were generally of low conductance, showed no voltage sensitivity. Steady-state and instantaneous Gj were constant with increasing Vj ≤ 40 mV. For higher Vjs, there was a slight but nonsignificant rise in steady-state Gj (Figures 4F, 4G, and 4I). Steady-state Gj of ShakB(L) homotypic channels declined with increasing Vjs (≥30 mV) of either polarity (Figures 4G, 4H, and 4J) [6]. Boltzmann parameters (Table S3) illustrate the symmetry of the response.

### Mechanism of Rectification at GFS Synapses

GFS heterotypic synapses modeled in *Xenopus* oocytes exhibited classical rectification. As observed at the crayfish giant synapse [1, 25, 26], depolarizations were preferentially transmitted in one direction only—in this case from the ShakB(N+16)-expressing cell (representing the presynaptic GF) to the ShakB(L)-expressing cell (representing the postsynaptic TTMn or PSI)—whereas hyperpolarizing signals passed preferentially in the opposite direction. Apart from this physiological asymmetry, notable features of the crayfish synaptic gap junctions are insensitivity to transmembrane voltage, very rapid responses to changes in transjunctional voltage, and steep rectification [25, 26]. With respect to the first of these, our data are consistent; conductance of ShakB(N+16)/ShakB(L) junctions was not significantly influenced by transmembrane voltage (Figure S3), and, hence, the observed voltage responses reflect Vj dependence only. The initial response to voltage was rapid, occurring within milliseconds of the imposition of Vj steps. At 5 ms (the earliest time at which we could reliably measure instantaneous conductance), the majority of voltage-sensitive channels had closed in response to hyperpolarizing Vjs applied to the ShakB(N+16)-expressing cell or depolarizing Vjs applied to the ShakB(L) cell. The rise to maximum Gj upon depolarization of the ShakB(N+16) cell, or hyperpolarization of the ShakB(L) cell, occurred more slowly so that, at 5 ms, Gj had only attained ∼50%–∼60% of its maximum value. These timescales are somewhat slower than those reported for the crayfish giant synapse, where junctional currents typically reached their steady-state levels within 1 ms of the application of Vj steps of ≤ 30 mV [25, 26]. ShakB heterotypic junctions showed steep rectification, albeit not as steep as that observed at the crayfish synapse. The Gjmin/Gjmax ratio was 0.21 (mean ± SEM, 0.2 ± 0.03 and 0.21 ± 0.01 when the ShakB(N+16) and ShakB(L) cells, respectively, were stepped) as compared to a corresponding ratio of the order of 0.05 for the crayfish giant synapse stepped over a similar Vj range [26].

Models of the crayfish giant motor synapse propose a structurally asymmetric junction in which one of the two apposed hemichannels contains a fast voltage-dependent gate [25, 26]. Qualitatively at least, the data presented here for the *Drosophila* GFS synapses are entirely consistent with this model. Given that ShakB(N+16) homotypic channels show little voltage sensitivity, the likely location of the voltage gate is postsynaptically in the ShakB(L) hemichannel. Assuming the crayfish synapses are composed of crustacean orthologs of ShakB, the quantitative differences between in situ- and in vitro-expressed junctions may be due to differences in the numbers and/or spatial arrangement of the channels in neurons and oocytes that might influence the kinetics of the voltage response.

### Conclusions

We have combined in vivo and in vitro approaches to characterize the molecular mechanism of transmission at putative rectifying electrical synapses in the *Drosophila* GFS. Studies in flies demonstrate that ShakB(N+16) in the presynaptic GF is necessary and sufficient to couple this cell to its postsynaptic targets TTMn and PSI, which express ShakB(L). *Xenopus* oocyte pairs in which ShakB(N+16) is expressed in one cell and ShakB(L) in the adjacent one form heterotypic channels that are asymmetrically gated by transjunctional voltage. Relative positivity of the ShakB(N+16)-expressing cell, or relative negativity of the ShakB(L)-expressing cell, leads to large junctional conductances and vice versa. Taken together, these data strongly support the hypothesis that differential voltage gating of structurally asymmetric gap junctions underlies rectification at arthropod electrical synapses.

## Supplemental Data

Document S1. Supplemental Results and Discussion, Three Figures, Three Tables, and Supplemental Experimental Procedures
