# Supplementary material for: Molecular Mechanism of Rectification at Identified Electrical Synapses in the Drosophila Giant Fiber System
Source: Curr Biol. 2008 Dec 23;18(24):1955–60. doi: 10.1016/j.cub.2008.10.067 (PMC2663713; doi:10.1016/j.cub.2008.10.067)
Supplement: Document S1. Supplemental Results and Discussion, Three Figures, Three Tables, and Supplemental Experimental Procedures [file mmc1.pdf]

Current Biology, *Volume 18*

Supplemental Data

Molecular Mechanism of Rectification at Identified Electrical Synapses in the *Drosophila* Giant Fiber System

Pauline Phelan, L. Ann Goulding, Jennifer L.Y. Tam, Marcus J. Allen, Rebecca J. Dawber, Jane A. Davies, and Jonathan P. Bacon

Supplemental Results and Discussion

Rescue of Synaptic Transmission in the GFS of *shakB*^{2} Mutants by Targeted Expression of *shakB* Transgenes

Table S1. Dye Coupling between Neurons of the Giant Fiber System

		Lucifer Yellow transfer to:		
Genotype	GFs dye filled (n)	GCIs	TTMn	PSI
Wild type	28	23 (82%)	17 (61%)	13 (46%)
shakB^2	15	0	0	0
A307, shakB(n+16)	32	18 (56%)	17 (53%)	2 (6%)
c17, shakB(n+16)	45	0	[a]	17 (38%)
A307, shakB(n)	11	0	0	0

[a]Possible weak coupling in 11/45 (24%) but cell identity could not be confirmed.

Genotypes of transgenic flies: *shakB*^{2}*; A307*-GAL4/UAS-*shakB*(*n+16)* [*A307, shakB(n+16)*]; *shakB*^{2}*; c17*-GAL4/UAS-*shakB*(*n+16*) or *shakB*^{2}*; c17*-GAL4/*c17*-GAL4; *UAS-shakB(n+16)* [*c17, shakB(n+16)*] and *shakB*^{2}*; A307*-GAL4*;* UAS-*shakB(n)* [*A307, shakB(n)*].

GF, Giant Fiber; GCI, giant commissural interneuron; TTMn, tergotrochanteral muscle motorneuron; PSI, peripherally synapsing interneuron

Table S2. Recordings of Synaptic Activity in the Giant Fiber System

		TTM			DLM
Genotype	n	Number responding	Response latency (ms ± SEM)	Responses to 10 stimuli at 100 Hz (± SEM)	Number responding
Control	7	7 (100%)	0.96 (± 0.03)	10.00 (± 0.00)	7 (100%)
shakB^2	36	8 (22%)	1.64 (± 0.07)	2.63 (± 0.92)	0
A307, shakB(n+16)	14	14 (100%)	1.45 (± 0.08)	6.20 (± 0.91)	2 (14%)
c17, shakB(n+16)	19	18 (95%)	1.65 (± 0.04)	5.50 (± 0.83)	0
A307, shakB(n)	12	9 (75%)	1.83 (± 0.09)	1.33 (± 0.17)	0

Intracellular recordings from the tergotrochanteral muscle (TTM) and dorsal longitudinal muscle (DLM) in response to stimulation of the Giant Fiber. n is the number recorded. Genotypes of transgenic flies are as listed in Table S1. Controls are *shakB*^{2}/+; UAS-*shakB(n+16)*; *shakB*^{2} are *shakB*^{2}/Y; UAS-*shakB(n+16)*. Parameters for controls and *shakB*^{2} mutants are similar to those previously reported [S13, S14].

Two methods, neuronal dye coupling and electrophysiological recordings of muscle activity, were used to assess the degree of rescue of synaptic transmission in the GFS of *shakB*^{2} mutants expressing UAS-*shakB* transgenes under the control of *A307*-GAL4 or *c17*-GAL4 (Figure 3; Tables S1 and S2). The electrophysiological approach provided a more sensitive measure of GF-TTMn conductivity than dye coupling in so far as it allowed the detection of synaptic transmission in *c17* transgenic flies that could not reliably be visualized by dye transfer. It was, however, less sensitive than dye transfer in detecting transmission through PSI. DLM responses were infrequently detected in *A307*, *shakB(n+16)* flies and never in *c17*, *shakB(n+16)* flies (Table S2) despite the presence of GF-PSI dye coupling (Figure 3H; Table S1). These data can be reconciled if one considers that the GF activates the TTM through a monosynaptic pathway and the DLMs through a disynaptic pathway. We have partially rescued first order synapses; this is sufficient in the monosynaptic pathway to drive muscle responses but is often inadequate in the longer disynaptic pathway that (given the lack of any DLM response in *shakB*^{2}; Table S2) seems to depend more heavily on electrical connections than the pathway to the TTM.

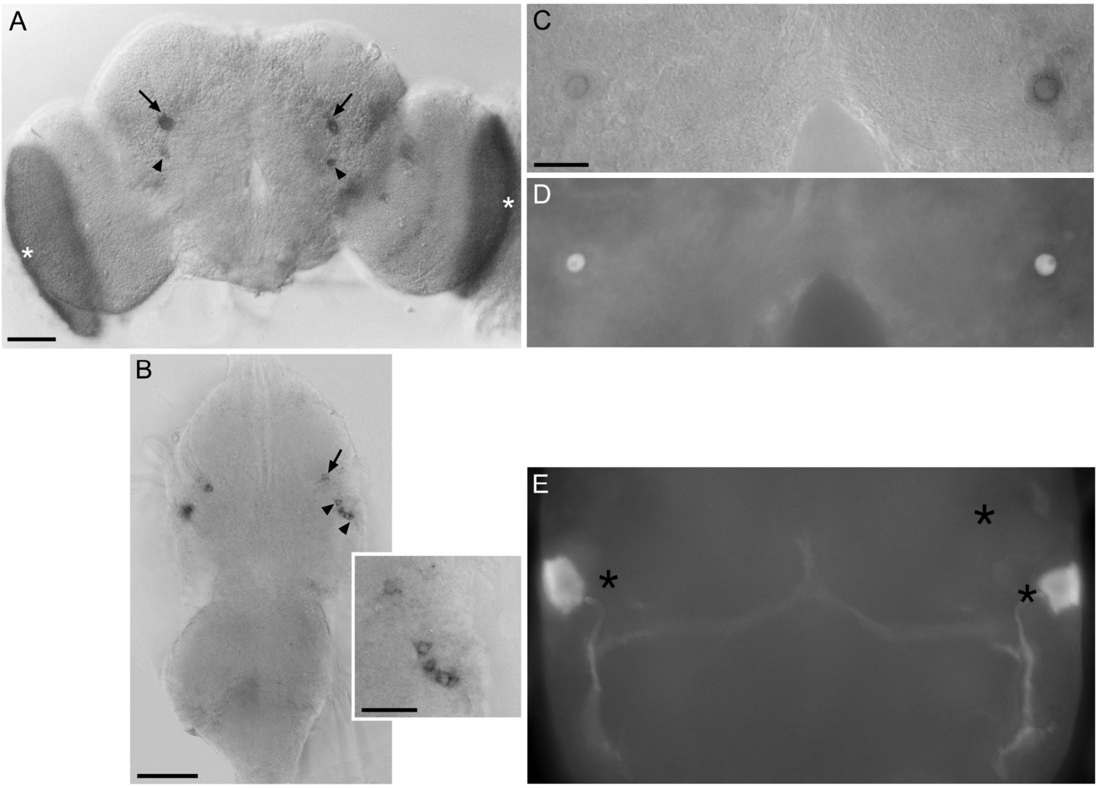

Figure S1. ***shakB(n+16)*** **Transcripts Are Expressed in the GF but Not in the TTMn**

Adult nervous systems, hybridized in situ with a DIG-labeled RNA probe that uniquely detects *shakB(n+16)* transcripts, were labeled with anti-DIG antibody or double labeled for DIG and reporter proteins expressed in the GF or TTMn. (A-B) Wild type, anti-DIG. In the brain (A) the *shakB(n+16)* RNA probe labels a large bilaterally symmetrical cell in the dorsal protocerebrum (arrows) believed to be the GF and small cells located just posterior (arrowheads) which approximate the position of the cell bodies of the GCIs. The optic lamina (asterisks) is also stained. In the thoracic ganglion (B), *shakB(n+16)* RNA is expressed in a cluster of (~ 3-4) cells (arrowheads; shown at higher magnification in the inset) located on each side of the mesothoracic ganglion and a single bilaterally symmetrical cell just anterior to these (arrow). (C-D) UAS-*lacZ*; *A307*-GAL4. *lacZ* expression marks the GFs. Double labeling with anti-DIG (C) and anti-β-gal (D) antibodies confirms that the large *shakB(n+16)*-expressing cells in the brain are the GFs. The ß-gal signal is significantly attenuated by the in situ hybridization procedure so that it is detectable only in the GF cell body (particularly the nuclear region) and not in the axons and dendrites. (E) w; *shakB(lethal)*-GAL4, UAS-*GFP*. GFP expression marks the TTMn. Preparations were double labeled with anti-DIG and anti-GFP antibodies and the images superimposed. Asterisks indicate the position of the DIG-labeled cell cluster and single more anterior cell (seen in B) relative to the GFP-labeled TTMn. The identity of the *shakB(n+16)*-expressing cells has not been established but none of these is TTMn. Scale bars A, B, 50 μm; B inset, 20 μm; C-E, 20 μm shown in C.

Functional Expression of ShakB Proteins in Paired *Xenopus* Oocytes

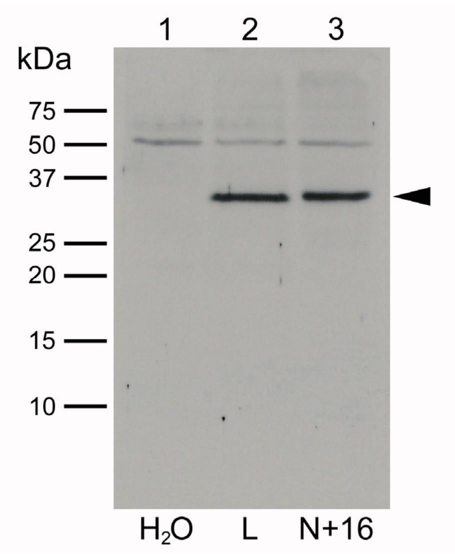

Figure S2. *shakB* RNAs Are Translated in *Xenopus* Oocytes Membrane fractions were prepared from oocytes ~36 hr after injection of in vitro transcribed *shakB* RNAs (10 ng) or water. Proteins (1-2 cell equivalents) were separated on SDS-polyacrylamide gels and transferred to nitrocellulose. The Western blot was probed with ShakB antisera. Labeled proteins of the expected molecular weight (~34 kDa; arrowhead) were detected in cells injected with *shakB(l)* (L, lane 2) or *shakB(n+16)* (N+16, lane 3) RNAs but not in water-injected control oocytes (H_2O; lane 1). Positions of molecular weight markers (kDa) are indicated on the left.

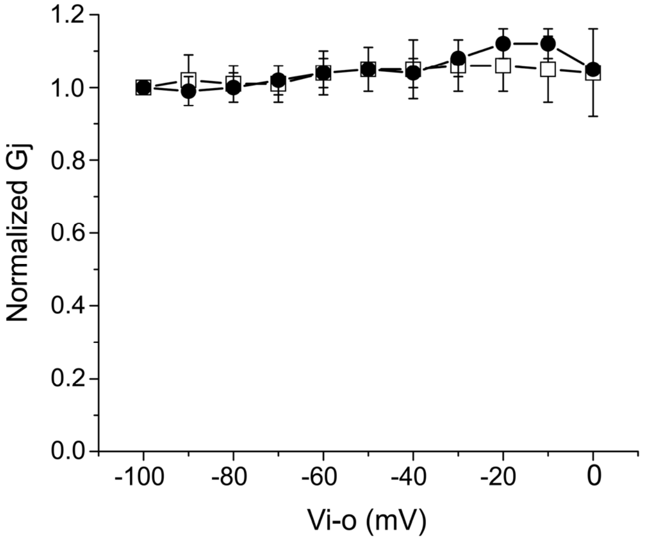

Figure S3. Conductance of ShakB(N+16)/ShakB(L) Heterotypic Channels Is Independent of Transmembrane Voltage

Xenopus oocytes injected with *shakB(n+16)* RNA were paired with oocytes injected with *shakB(l)* RNA (0.1-0.5 ng). Cell pairs were recorded 24-48 hr later by dual voltage clamp. Both oocytes of a pair were clamped simultaneously to a series of holding potentials between -100 and 0 mV to establish an inside-outside potential (Vi-o). At each potential, brief depolarizing or hyperpolarizing pulses (10 mV, 250 ms) were applied to the ShakB(N+16)-expressing cell (open squares, □) or the ShakB(L)-expressing cell (filled circles, ●) and junctional current was recorded in the neighboring cell of the pair. The calculated conductance (Gj) was normalized to its value at -100 mV. Values are mean ± SD for n = 7 (□) and 6 (●) except 0 mV where n = 5 (□) and 1 (●). Data are shown for depolarizing pulses. Application of hyperpolarizing pulses yielded the same results.

Table S3. Functional Expression of ShakB Proteins in Paired *Xenopus* Oocytes

	[a]Junctional Conductance (Gj)			[b]Boltzmann Parameters			
Cell 1 / Cell 2	Mean at +Vj (µS ± SEM)	Mean at -Vj (µS ± SEM)	n	Gjmax (µS ± SD)	Gjmin (µS ± SD)	V_0 (mV ± SD)	n
Heterotypic							
N+16 / L	4.39 ± 0.52	2.78 ± 0.36	49	3.09 ± 0.23	0.59 ± 0.07	6.01 ± 3.21	4
L / N+16	3.52 ± 0.44	5.34 ± 0.55	49	2.7 ± 0.26	0.51 ± 0.04	-3.63 ± 5.37	4
Homotypic							
N+16 / N+16	2.36 ± 0.36	2.38 ± 0.36	26	no significant voltage sensitivity			
L / L	7.64 ± 1.74	7.73 ± 1.77	26	1.01 ± 0.03 (+)	0.40 ± 0.03 (+)	36.43 ± 1.88 (+)	3
				1.00 ± 0.08 (-)	0.41 ± 0.04 (-)	-34.34 ± 3.19 (-)	

[a]Both cells were clamped to a holding potential of -80 mV. Depolarizing (+Vj) or hyperpolarizing (-Vj) voltage steps were applied to Cell 1 while the current (Ij) required to maintain Cell 2 at the holding potential was recorded. Gj is Ij/Vj. Values are mean for n cell pairs injected with 0.1-0.5 ng RNA (heterotypic), 0.25-2 ng (N+16 homotypic) and 0.05-0.25 ng (L homotypic).

[b]Graphs of steady state Gj versus Vj are shown in Figures 4D and 4E (heterotypic) and Figures 4I and 4J (homotypic). These data were fitted to a Boltzmann equation as described in Experimental Procedures. For heterotypic pairs, data were fitted to a single sigmoid; for homotypic pairs, separate curves were fitted for positive (+) and negative (-) Vjs. Gjmax and Gjmin are, respectively, maximum and minimum normalized Gj. V_0 is the Vj at which Gj is halfway between Gjmin and Gjmax. n is the number of cell pairs.

N+16: ShakB(Neural+16); L: ShakB(Lethal)

Supplemental Experimental Procedures

Drosophila Stocks

Wild-type strains were Oregon-R or Berlin. *shakB*^{2} is an X-chromosome mutation that eliminates *shakB(n)* and *shakB(n+16)* function [S1, S2]. P[GAL4]*A307* and P[GAL4]*c17* drive transgene expression in the GFS [S3-S5]. UAS-*shakB(n)* and UAS-*shakB(n+16)* carry P-element insertions of *shakB(n)* and *shakB(n+16),* respectively, fused to a GAL4-sensitive promoter (UAS). To rescue *shakB* expression in the GFS of *shakB*^{2} mutants, UAS and/or GAL4 lines were crossed into a mutant background. These lines were then used to generate flies of the following genotypes: *shakB*^{2}*; A307*-GAL4/UAS-*shakB*(*n+16)* [*A307*, *shakB*(*n+16)*], *shakB*^{2}*; c17*-GAL4/UAS-*shakB*(*n+16*) or *shakB*^{2}*; c17*-GAL4/*c17*-GAL4; UAS-*shakB*(*n+16*) [*c17*, *shakB*(*n+16)*] and *shakB*^{2}*; A307*-GAL4*;* UAS-*shakB(n)* [*A307*, *shakB(n)*].

mRNA In Situ Hybridization to *Drosophila* Nervous Systems

A digoxigenin (DIG)-labeled RNA probe corresponding to a unique sequence of the *shakB(n+16)* transcript (nucleotides 539-874 of the N3 sequence) [S2] was synthesized using a DIG-labeling kit (Boehringer Mannheim) according to the manufacturer's instructions. The probe was hybridized in situ to adult nervous systems and detected with anti-DIG antibody (Boehringer Mannheim or Roche; 1:2000) using a modification of the method of Tautz and Pfeifle [S6, S7]. To identify *shakB(n+16)*-expressing cells, nervous systems of UAS-*lacZ*; *A307*-GAL4 or w; *shakB(lethal)*-GAL4, UAS-*GFP* (gift, T.A. Godenschwege) flies were double labeled for RNA and β-galactosidase (β-gal) or GFP reporter proteins, respectively. In situ hybridization and detection with anti-DIG antibody were performed first followed by immunofluorescent labeling with anti-β-gal or anti-GFP antibodies using the general methods described below.

Immunofluorescent Labeling of *Drosophila* Nervous Systems

Primary antibodies: rabbit anti-ShakB (1:50), raised to a C-terminal peptide common to all ShakB proteins [S3], rabbit anti-Lucifer Yellow (Molecular Probes; 1:100-1:500), rabbit anti-β-galactosidase (Cappel; 1:5000), rabbit anti-GFP (Santa Cruz; 1:500). Secondary antibodies: Alexa Fluor-conjugated goat anti-rabbit (Molecular Probes; 1:500), Cy5 donkey anti-rabbit (Stratech; 1:100). Nervous systems were dissected in *Drosophila* saline [S3], fixed in 4% paraformaldehyde in phosphate buffered saline (PBS) for 30 min at room temperature and washed in PBS. After pre-incubation in blocking solution: 0.1M L-lysine (or alternatively 1% BSA; Sigma), 4% normal goat serum (Dako) in PBS containing 0.1-0.5% Triton X-100 (PBT-X), preparations were incubated overnight at 4˚C in primary antibodies diluted in blocking solution. Preparations were then washed in PBT-X, 6 x 10 min, incubated in secondary fluorescent antibodies (in blocking solution) for 2 hr at room temperature and washed as before. Labeled preparations were dehydrated through a glycerol or ethanol series and slide-mounted in Vectashield (Vector Laboratories) or methylsalicylate, respectively.

Intracellular Injection of Lucifer Yellow in the GFS

Nervous systems were dissected in cold *Drosophila* saline and mounted on poly-l-lysine (0.1% w/v)-coated glass coverslips in a chamber containing fresh saline. The chamber was transferred to the stage of a microscope equipped with fluorescence and Nomarski water-immersion objectives. One GF axon in the cervical connective was impaled with a Lucifer Yellow-containing glass microelectrode and dye filled as previously described [S3].

Microscopy and Image Analysis

DIG labeled preparations were viewed and photographed under Nomarski or fluorescence optics with Zeiss Axiophot or Leica DMR microscopes. All other images are projections of confocal z series taken at 1 μm steps with Zeiss LSM 510 or Leica TCS SP2 confocal microscopes and associated software. Images were processed in Adobe Photoshop 6.0.

GFS Electrophysiology

Intracellular recordings of activity in the TTM and DLM muscles were made in response to extracellular activation of the GFs as described [S5].

Functional Expression of In Vitro Transcribed RNAs in Paired *Xenopus* Oocytes

shakB RNAs were transcribed in vitro as described [S8]. Stage V-VI *Xenopus laevis* oocytes were isolated and defolliculated as described [S9]. Oocytes were cultured in Barth's medium [S10] with high Ca^{++} (3 mM) to reduce hemichannel activity. Defolliculated cells were pre-injected (Nanoject injector; Drummond) with *Xenopus* connexin 38 DNA antisense oligonucleotide (20 ng) [S8] to eliminate endogenous coupling. *shakB* RNAs (0.05-2 ng) in RNase-free H_2O, or H_2O only as a control, were injected 18-24 hr later. The vitelline envelope was removed after brief incubation in hypertonic medium [S11] and cells were paired with the vegetal membranes apposed. After incubation for 24-48 hr the formation of intercellular channels was recorded by dual voltage clamp [S12]. Each cell of a pair was impaled with two borosilicate glass electrodes filled with recording solution [S8]. Voltage clamp was carried out with two GeneClamp500 amplifiers interfaced to a PC via Digidata 1320A and data were recorded and analyzed using pClamp 8.0 software (Axon Instruments). The relationship between voltage and junctional conductance was quantified by fitting the data (Origin 7 software, OriginLab) to a Boltzmann equation of the form $y = A2 + A1-A2/(1+\exp((x-x0)/dx))$ where A1 and A2 are maximum and minimum conductance, respectively, x0 is the voltage at which conductance is halfway between its maximum and minimum values and dx represents the change in conductance over the voltage range, a measure of voltage sensitivity.

Gel Electrophoresis and Western Blotting of Oocyte Membrane Fractions

Xenopus oocytes microinjected with *shakB* RNAs were fractionated using the 'sucrose-cushion' method of Colman [S10]. Membrane proteins were electrophoretically separated on 12.5% SDS-polyacrylamide gels and transferred to nitrocellulose (Hybond-ECL; Amersham). Western blots were labeled with anti-ShakB antisera (1:100) as described [S8].

Supplemental References

S1. Krishnan, S.N., Frei, E., Swain, G.P., and Wyman, R.J. (1993). *Passover*: A gene required for synaptic connectivity in the Giant Fiber System of Drosophila. Cell *73*, 967-977.

S2. Zhang, Z., Curtin, K.D., Sun, Y.-A., and Wyman, R.J. (1999). Nested transcripts of gap junction gene have distinct expression patterns. J. Neurobiol. *40*, 288-301.

S3. Phelan, P., Nakagawa, M., Wilkin, M.B., Moffat, K.G., O'Kane, C.J., Davies, J.A., and Bacon, J.P. (1996). Mutations in *shaking-B* prevent electrical synapse formation in the *Drosophila* Giant Fiber System. J. Neurosci. *16*, 1101-1113.

S4. Allen, M.J., Drummond, J.A., and Moffat, K.G. (1998). Development of the Giant Fiber neuron of *Drosophila melanogaster.* J. Comp. Neurol. *397*, 519-531.

S5. Allen M.J., Shan X., Caruccio P., Froggett S.J., Moffat K.G., and Murphey R.K. (1999). Targeted expression of truncated Glued disrupts Giant Fiber synapse formation in *Drosophila*. J. Neurosci. *19*, 9374-9384.

S6. Tautz, D., and Pfeifle, C. (1989). A non-radioactive *in situ* hybridization method for the localization of specific RNAs in *Drosophila* embryos reveals translational control of the segmentation gene *hunchback*. Chromosoma *98*, 81-85.

S7. Crompton, D., Todman, M., Wilkin, M., Ji, S., and Davies, J. (1995). Essential and neural transcripts from the *Drosophila shaking-B* locus are differentially expressed in the embryonic mesoderm and pupal nervous system. Dev. Biol. *170*, 142-158.

S8. Phelan, P., Stebbings, L.A., Baines, R.A., Bacon, J.P., Davies, J.A., and Ford, C. (1998). *Drosophila* Shaking-B protein forms gap junctions in paired *Xenopus* oocytes. Nature *391*, 181-184.

S9. Swenson, K.I., Jordan, J.R., Beyer, E.C., and Paul. D.L. (1989). Formation of gap junctions by expression of connexins in Xenopus oocyte pairs. Cell *57*, 145-155.

S10. Colman, A. (1984). Expression of exogenous RNA in *Xenopus* oocytes. In Transcription and Translation- a practical approach, B.D. Hames, S.J. Higgins, ed. (IRL, Oxford) pp. 271-302.

S11. Methfessel, C., Witzemann, V., Takahashi, T., Mishina, M., Numa, S., and Sakmann, B. (1986). Patch clamp measurements on Xenopus laevis oocytes: currents through endogenous channels and implanted acetylcholine receptor and sodium channels. Pflugers Arch. *407*, 577-588.

S12. Spray, D.C., Harris, A.L., and Bennett, M.V.L. (1981). Equilibrium properties of a voltage-dependent junctional conductance. J. Gen. Physiol. *77*, 77-93.

S13. Thomas, J.B., and Wyman, R.J. (1984). Mutations altering synaptic connectivity between identified neurons in *Drosophila.* J. Neurosci. *4*, 530-538.

S14. Baird, D.H., Schalet, A.P., and Wyman, R.J. (1990). The *Passover* locus in *Drosophila melanogaster*: complex complementation and different effects on the Giant Fiber neural pathway. Genetics *126*, 1045-1059.
